# Supplementary material for: Clinical profile and factors associated with COVID-19 in Yaounde, Cameroon: A prospective cohort study
Source: PLoS One. 2021 May 12;16(5):e0251504. doi: 10.1371/journal.pone.0251504 (PMC8115782; doi:10.1371/journal.pone.0251504)
Supplement: S1 File — (DOCX) [file pone.0251504.s001.docx]

> Sexetable=table(sev$severe, sev$Sexe)

> oddsratio(t(Sexetable), method = "wald")

$data

0 1 Total

F 81 8 89

M 132 30 162

Total 213 38 251

$measure

odds ratio with 95% C.I.

estimate lower upper

F 1.000000 NA NA

M 2.301136 1.005919 5.264069

$p.value

two-sided

midp.exact fisher.exact chi.square

F NA NA NA

M 0.04224074 0.0450647 0.04389763

$correction

[1] FALSE

attr(,"method")

[1] "Unconditional MLE & normal approximation (Wald) CI"

> agegrouptable=table(sev$severe, sev$agegroup)

> oddsratio(t(agegrouptable), method = "wald")

$data

0 1 Total

0-17 8 0 8

18-29 36 0 36

30-39 54 5 59

40-49 37 10 47

50-59 38 10 48

60-69 26 12 38

70+ 14 1 15

Total 213 38 251

$measure

odds ratio with 95% C.I.

estimate lower upper

0-17 1 NA NA

18-29 NaN NaN NaN

30-39 Inf NaN Inf

40-49 Inf NaN Inf

50-59 Inf NaN Inf

60-69 Inf NaN Inf

70+ Inf NaN Inf

$p.value

two-sided

midp.exact fisher.exact chi.square

0-17 NA NA NA

18-29 1.00000000 1.00000000 NaN

30-39 0.51838564 1.00000000 0.39202854

40-49 0.17703610 0.32625939 0.14920320

50-59 0.18369159 0.32646417 0.15432359

60-69 0.06958191 0.08983661 0.06449079

70+ 0.65217391 1.00000000 0.45523866

$correction

[1] FALSE

attr(,"method")

[1] "Unconditional MLE & normal approximation (Wald) CI"

Warning messages:

1: In chisq.test(xx, correct = correction) :

Chi-squared approximation may be incorrect

2: In chisq.test(xx, correct = correction) :

Chi-squared approximation may be incorrect

3: In chisq.test(xx, correct = correction) :

Chi-squared approximation may be incorrect

4: In chisq.test(xx, correct = correction) :

Chi-squared approximation may be incorrect

5: In chisq.test(xx, correct = correction) :

Chi-squared approximation may be incorrect

6: In chisq.test(xx, correct = correction) :

Chi-squared approximation may be incorrect

> Professiontable=table(sev$severe, sev$Profession)

> oddsratio(t(Professiontable), method = "wald")

$data

0 1 Total

Employed (formal) 48 10 58

Employed (informal) 76 19 95

Health professionals 19 0 19

Prisoners 11 0 11

Religious leaders 1 0 1

Retired 31 9 40

Student 23 0 23

Unemployed 4 0 4

Total 213 38 251

$measure

odds ratio with 95% C.I.

estimate lower upper

Employed (formal) 1.000000 NA NA

Employed (informal) 1.200000 0.5145924 2.798331

Health professionals 0.000000 0.0000000 NaN

Prisoners 0.000000 0.0000000 NaN

Religious leaders 0.000000 0.0000000 NaN

Retired 1.393548 0.5088609 3.816322

Student 0.000000 0.0000000 NaN

Unemployed 0.000000 0.0000000 NaN

$p.value

two-sided

midp.exact fisher.exact chi.square

Employed (formal) NA NA NA

Employed (informal) 0.6863119 0.83204615 0.67274745

Health professionals 0.0475659 0.05959556 0.05234222

Prisoners 0.1534544 0.34546992 0.13640956

Religious leaders 0.8305085 1.00000000 0.64865482

Retired 0.5283597 0.60591752 0.51750249

Student 0.0277788 0.05506403 0.03342181

Unemployed 0.4853051 1.00000000 0.36451405

$correction

[1] FALSE

attr(,"method")

[1] "Unconditional MLE & normal approximation (Wald) CI"

Warning messages:

1: In chisq.test(xx, correct = correction) :

Chi-squared approximation may be incorrect

2: In chisq.test(xx, correct = correction) :

Chi-squared approximation may be incorrect

3: In chisq.test(xx, correct = correction) :

Chi-squared approximation may be incorrect

4: In chisq.test(xx, correct = correction) :

Chi-squared approximation may be incorrect

5: In chisq.test(xx, correct = correction) :

Chi-squared approximation may be incorrect

> Diarrhéestable=table(sev$severe, sev$Diarrhées)

> oddsratio(t(Diarrhéestable), method = "wald")

$data

0 1 Total

NON 159 23 182

OUI 54 15 69

Total 213 38 251

$measure

odds ratio with 95% C.I.

estimate lower upper

NON 1.00000 NA NA

OUI 1.92029 0.9346954 3.94515

$p.value

two-sided

midp.exact fisher.exact chi.square

NON NA NA NA

OUI 0.08350244 0.0788166 0.07247155

$correction

[1] FALSE

attr(,"method")

[1] "Unconditional MLE & normal approximation (Wald) CI"

> Grossessetable=table(sev$severe, sev$Grossesse)

> oddsratio(t(Grossessetable), method = "wald")

$data

0 1 Total

NON 209 38 247

OUI 4 0 4

Total 213 38 251

$measure

odds ratio with 95% C.I.

estimate lower upper

NON 1 NA NA

OUI 0 0 NaN

$p.value

two-sided

midp.exact fisher.exact chi.square

NON NA NA NA

OUI 0.5163598 1 0.3944528

$correction

[1] FALSE

attr(,"method")

[1] "Unconditional MLE & normal approximation (Wald) CI"

Warning message:

In chisq.test(xx, correct = correction) :

Chi-squared approximation may be incorrect

> vihtable=table(sev$severe, sev$vih)

> oddsratio(t(vihtable), method = "wald")

$data

0 1 Total

NON 209 35 244

OUI 4 3 7

Total 213 38 251

$measure

odds ratio with 95% C.I.

estimate lower upper

NON 1.000000 NA NA

OUI 4.478571 0.9609361 20.87298

$p.value

two-sided

midp.exact fisher.exact chi.square

NON NA NA NA

OUI 0.08401641 0.07273238 0.03797671

$correction

[1] FALSE

attr(,"method")

[1] "Unconditional MLE & normal approximation (Wald) CI"

Warning message:

In chisq.test(xx, correct = correction) :

Chi-squared approximation may be incorrect

> Hypertable=table(sev$severe, sev$Hypertension)

> oddsratio(t(Hypertable), method = "wald")

$data

0 1 Total

NON 177 25 202

OUI 36 13 49

Total 213 38 251

$measure

odds ratio with 95% C.I.

estimate lower upper

NON 1.000000 NA NA

OUI 2.556667 1.195694 5.466736

$p.value

two-sided

midp.exact fisher.exact chi.square

NON NA NA NA

OUI 0.02039397 0.02401957 0.01314497

$correction

[1] FALSE

attr(,"method")

[1] "Unconditional MLE & normal approximation (Wald) CI"

> Diabètetable=table(sev$severe, sev$Diabète)

> oddsratio(t(Diabètetable), method = "wald")

$data

0 1 Total

NON 204 32 236

OUI 9 6 15

Total 213 38 251

$measure

odds ratio with 95% C.I.

estimate lower upper

NON 1.00 NA NA

OUI 4.25 1.417312 12.74419

$p.value

two-sided

midp.exact fisher.exact chi.square

NON NA NA NA

OUI 0.01721987 0.01447017 0.005600175

$correction

[1] FALSE

attr(,"method")

[1] "Unconditional MLE & normal approximation (Wald) CI"

Warning message:

In chisq.test(xx, correct = correction) :

Chi-squared approximation may be incorrect

> oxygenetable=table(sev$severe, sev$oxygene)

> oddsratio(t(oxygenetable), method = "wald")

$data

0 1 Total

NON 213 2 215

OUI 0 36 36

Total 213 38 251

$measure

odds ratio with 95% C.I.

estimate lower upper

NON 1 NA NA

OUI Inf NaN Inf

$p.value

two-sided

midp.exact fisher.exact chi.square

NON NA NA NA

OUI 0 1.495076e-41 3.622841e-53

$correction

[1] FALSE

attr(,"method")

[1] "Unconditional MLE & normal approximation (Wald) CI"

> dysptable=table(sev$severe, sev$dyspnediff)

> oddsratio(t(dysptable), method = "wald")

$data

0 1 Total

NON 115 7 122

OUI 98 31 129

Total 213 38 251

$measure

odds ratio with 95% C.I.

estimate lower upper

NON 1.000000 NA NA

OUI 5.196793 2.191824 12.32155

$p.value

two-sided

midp.exact fisher.exact chi.square

NON NA NA NA

OUI 4.002275e-05 4.082034e-05 5.315346e-05

$correction

[1] FALSE

attr(,"method")

[1] "Unconditional MLE & normal approximation (Wald) CI"

> maladiepultable=table(sev$severe, sev$maladiepul)

> oddsratio(t(maladiepultable), method = "wald")

$data

0 1 Total

NON 210 35 245

OUI 3 3 6

Total 213 38 251

$measure

odds ratio with 95% C.I.

estimate lower upper

NON 1 NA NA

OUI 6 1.164086 30.92556

$p.value

two-sided

midp.exact fisher.exact chi.square

NON NA NA NA

OUI 0.05182918 0.04639737 0.01589464

$correction

[1] FALSE

attr(,"method")

[1] "Unconditional MLE & normal approximation (Wald) CI"

Warning message:

In chisq.test(xx, correct = correction) :

Chi-squared approximation may be incorrect

> cardiotable=table(sev$severe, sev$cardio)

> oddsratio(t(cardiotable), method = "wald")

$data

0 1 Total

NON 198 32 230

OUI 15 6 21

Total 213 38 251

$measure

odds ratio with 95% C.I.

estimate lower upper

NON 1.000 NA NA

OUI 2.475 0.894495 6.848138

$p.value

two-sided

midp.exact fisher.exact chi.square

NON NA NA NA

OUI 0.1009848 0.103887 0.0728181

$correction

[1] FALSE

attr(,"method")

[1] "Unconditional MLE & normal approximation (Wald) CI"

Warning message:

In chisq.test(xx, correct = correction) :

Chi-squared approximation may be incorrect
