# Supplementary material for: Clinical profile and factors associated with COVID-19 in Yaounde, Cameroon: A prospective cohort study
Source: PLoS One. 2021 May 12;16(5):e0251504. doi: 10.1371/journal.pone.0251504 (PMC8115782; doi:10.1371/journal.pone.0251504)
Supplement: S2 File — (DOCX) [file pone.0251504.s002.docx]

**Comorbidity**

> print(res1d)

glmulti.analysis

Method: h / Fitting: glm / IC used: aicc

Level: 1 / Marginality: FALSE

From 100 models:

Best IC: 176.290745704018

Best model:

[1] "severe ~ 1 + Sexe + vih + Diabète + maladiepul + agegroup_40_49 + "

[2] " agegroup_50_59 + agegroup_60_69"

Evidence weight: 0.0474967909681037

Worst IC: 180.587598438377

8 models within 2 IC units.

91 models to reach 95% of evidence weight.


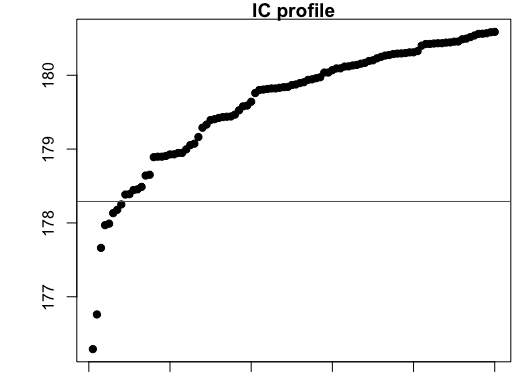


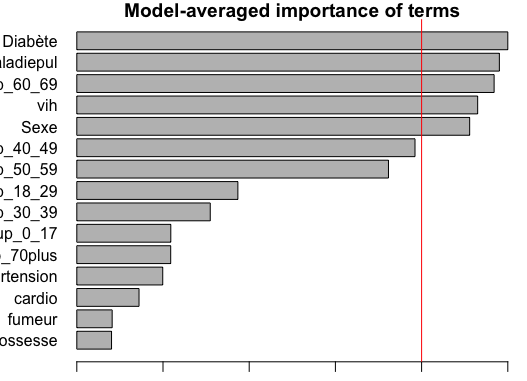


> top <- weightable(res1d)

> top <- top[top$aicc <= min(top$aicc) + 2,]

> top

model

1 severe ~ 1 + Sexe + vih + Diabète + maladiepul + agegroup_40_49 + agegroup_50_59 + agegroup_60_69

2 severe ~ 1 + Sexe + vih + Diabète + maladiepul + agegroup_30_39 + agegroup_40_49 + agegroup_50_59 + agegroup_60_69

3 severe ~ 1 + Sexe + vih + Diabète + maladiepul + agegroup_18_29 + agegroup_40_49 + agegroup_50_59 + agegroup_60_69

4 severe ~ 1 + Sexe + vih + Hypertension + Diabète + maladiepul + agegroup_40_49 + agegroup_50_59 + agegroup_60_69

5 severe ~ 1 + Sexe + vih + Diabète + maladiepul + cardio + agegroup_40_49 + agegroup_50_59 + agegroup_60_69

6 severe ~ 1 + Sexe + vih + Diabète + maladiepul + agegroup_0_17 + agegroup_40_49 + agegroup_50_59 + agegroup_60_69

7 severe ~ 1 + vih + Diabète + maladiepul + agegroup_40_49 + agegroup_50_59 + agegroup_60_69

8 severe ~ 1 + Sexe + vih + Hypertension + Diabète + maladiepul + agegroup_30_39 + agegroup_40_49 + agegroup_50_59 + agegroup_60_69

aicc weights

1 176.2907 0.04749679

2 176.7603 0.03755764

3 177.6618 0.02392999

4 177.9697 0.02051541

5 177.9903 0.02030545

6 178.1323 0.01891355

7 178.1777 0.01848887

8 178.2507 0.01782646

> summary(res1d@objects[[1]])

Call:

fitfunc(formula = as.formula(x), data = data)

Deviance Residuals:

Min 1Q Median 3Q Max

-0.52788 -0.20825 -0.06927 0.01988 0.93073

Coefficients:

Estimate Std. Error t value Pr(>|t|)

(Intercept) -0.01988 0.04129 -0.481 0.630650

SexeM 0.08915 0.04490 1.985 0.048226 *

vihOUI 0.27434 0.13080 2.097 0.036995 *

DiabèteOUI 0.22961 0.09058 2.535 0.011877 *

maladiepulOUI 0.31892 0.13967 2.283 0.023274 *

agegroup_40_49 0.13898 0.05854 2.374 0.018369 *

agegroup_50_59 0.12586 0.05816 2.164 0.031457 *

agegroup_60_69 0.22884 0.06357 3.599 0.000386 ***

---

Signif. codes: 0 ‘***’ 0.001 ‘**’ 0.01 ‘*’ 0.05 ‘.’ 0.1 ‘ ’ 1

(Dispersion parameter for gaussian family taken to be 0.1132881)

Null deviance: 32.247 on 250 degrees of freedom

Residual deviance: 27.529 on 243 degrees of freedom

AIC: 175.54

Number of Fisher Scoring iterations: 2

> par("mar")

[1] 1 1 1 1

> par(mar=c(1,1,1,1))

> plot(res1d, type="s")

> bestmodeldum<-glm(severe ~ Sexe + vih + Diabète + maladiepul + agegroup_40_49 + agegroup_50_59 + agegroup_60_69, data = sevdum, family = "binomial")

> exp(cbind(OR = coef(bestmodeldum), confint(bestmodeldum)))

Waiting for profiling to be done...

OR 2.5 % 97.5 %

(Intercept) 0.02332267 0.006729643 0.06442429

SexeM 2.52942525 1.061759513 6.76530837

vihOUI 5.57758169 0.924079535 31.76018297

DiabèteOUI 4.05551851 1.122660603 14.15557955

maladiepulOUI 6.29717838 0.909753762 44.93456456

agegroup_40_49 4.54410687 1.519373062 14.63373318

agegroup_50_59 4.09797208 1.363703283 13.19942983

agegroup_60_69 7.41892107 2.522447902 23.85523153

**Symptoms**

Due to limited computation capabilities gmulti algoritms couldn’t completely be applied in this case whereby we have almost 25 variables to consider in the models.

> bestmodelssd<-glm(severe~Sexe+dyspnediff+Fatigue+doulabdo+doulmusc+agegroup_40_49+agegroup_50_59+agegroup_60_69, data = sevdum, family = "binomial")

> exp(cbind(OR = coef(bestmodelssd), confint(bestmodelssd)))

Waiting for profiling to be done...

OR 2.5 % 97.5 %

(Intercept) 0.004046981 0.0006635577 0.01797531

SexeM 2.750810798 1.1242226690 7.40585323

dyspnediffOUI 3.705554640 1.4623007098 10.45438833

FatigueOUI 3.357287118 1.2606982275 10.12907155

doulabdoOUI 0.319134108 0.0473496319 1.27500568

doulmuscOUI 2.252151258 1.0028536077 5.23412696

agegroup_40_49 4.799468047 1.4962857830 16.63314895

agegroup_50_59 3.198721481 1.0131076878 10.79922502

agegroup_60_69 6.505621556 2.0788267603 22.30270909

> #exp(coef(bestmodel))

> summary(bestmodelssd)

Call:

glm(formula = severe ~ Sexe + dyspnediff + Fatigue + doulabdo +

doulmusc + agegroup_40_49 + agegroup_50_59 + agegroup_60_69,

family = "binomial", data = sevdum)

Deviance Residuals:

Min 1Q Median 3Q Max

-1.4888 -0.5465 -0.2709 -0.1488 2.7243

Coefficients:

Estimate Std. Error z value Pr(>|z|)

(Intercept) -5.5098 0.8365 -6.587 4.49e-11 ***

SexeM 1.0119 0.4764 2.124 0.03368 *

dyspnediffOUI 1.3098 0.4959 2.641 0.00826 **

FatigueOUI 1.2111 0.5239 2.312 0.02080 *

doulabdoOUI -1.1421 0.8002 -1.427 0.15348

doulmuscOUI 0.8119 0.4190 1.938 0.05267 .

agegroup_40_49 1.5685 0.6063 2.587 0.00968 **

agegroup_50_59 1.1628 0.5954 1.953 0.05083 .

agegroup_60_69 1.8727 0.5982 3.131 0.00174 **

---

Signif. codes: 0 ‘***’ 0.001 ‘**’ 0.01 ‘*’ 0.05 ‘.’ 0.1 ‘ ’ 1

(Dispersion parameter for binomial family taken to be 1)

Null deviance: 213.41 on 250 degrees of freedom

Residual deviance: 160.82 on 242 degrees of freedom

AIC: 178.82

Number of Fisher Scoring iterations: 6
