# Supplementary material for: Clinical profile and factors associated with COVID-19 in Yaounde, Cameroon: A prospective cohort study
Source: PLoS One. 2021 May 12;16(5):e0251504. doi: 10.1371/journal.pone.0251504 (PMC8115782; doi:10.1371/journal.pone.0251504)
Supplement: S3 File — (DOCX) [file pone.0251504.s003.docx]

**Model for risk prediction of COVID-19 Diagnosis**


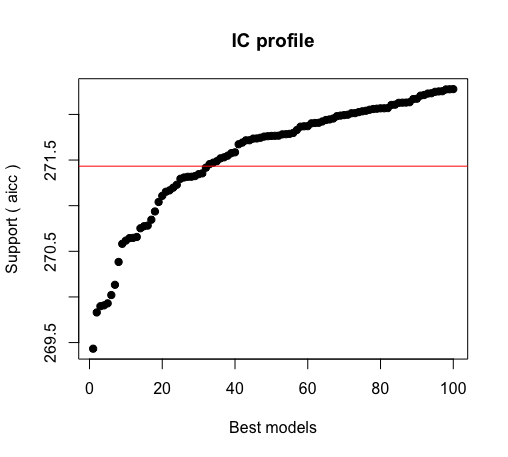


> print(resdiag)

**glmulti.analysis**

Method: h / Fitting: glm / IC used: aicc

Level: 1 / Marginality: FALSE

From 100 models:

Best IC: 269.432851207074

**Best model:**

**[1] "Covid19 ~ 1 + Frissons + Fatigue + doulabdo + doulmusc + nezcoule + "**

**[2] " gout + rassemblement"**

Evidence weight: 0.027172755422282

Worst IC: 272.277402626645

32 models within 2 IC units.

92 models to reach 95% of evidence weight.

> **top**

**model**

1 Covid19 ~ 1 + Frissons + Fatigue + doulabdo + doulmusc + nezcoule + gout + rassemblement

2 Covid19 ~ 1 + Frissons + Fatigue + doulabdo + doulmusc + nezcoule + gout + fumeur + rassemblement

3 Covid19 ~ 1 + Profession + Frissons + Fatigue + doulabdo + doulmusc + nezcoule + gout + rassemblement

4 Covid19 ~ 1 + agegroup + Profession + Frissons + Fatigue + doulabdo + doulmusc + nezcoule + gout + rassemblement

5 Covid19 ~ 1 + Frissons + Fatigue + doulabdo + doulmusc + nezpris + nezcoule + gout + rassemblement

aicc weights

1 269.4329 0.02717276

2 269.8300 0.02227837

3 269.8983 0.02153075

4 269.9078 0.02142942

5 269.9298 0.02119471

> **summary(resdiag@objects[[1]])**

Call:

fitfunc(formula = as.formula(x), data = data)

Deviance Residuals:

Min 1Q Median 3Q Max

-0.94677 -0.00872 0.10185 0.20035 0.41266

Coefficients:

Estimate Std. Error t value Pr(>|t|)

(Intercept) 0.79965 0.03780 21.153 <2e-16 ***

FrissonsOUI -0.10483 0.05032 -2.083 0.0381 *

FatigueOUI -0.10748 0.04518 -2.379 0.0180 *

doulabdoOUI 0.14712 0.07270 2.024 0.0439 *

doulmuscOUI 0.11057 0.04473 2.472 0.0140 *

nezcouleOUI -0.18787 0.08071 -2.328 0.0206 *

goutOUI 0.09850 0.04291 2.295 0.0224 *

rassemblementOUI 0.11768 0.05542 2.124 0.0345 *

---

Signif. codes: 0 ‘***’ 0.001 ‘**’ 0.01 ‘*’ 0.05 ‘.’ 0.1 ‘ ’ 1

(Dispersion parameter for gaussian family taken to be 0.1339105)

Null deviance: 44.684 on 312 degrees of freedom

Residual deviance: 40.843 on 305 degrees of freedom

AIC: 268.84

Number of Fisher Scoring iterations: 2

**> exp(cbind(OR = coef(bestmodel), confint(bestmodel)))**

Waiting for profiling to be done...

OR 2.5 % 97.5 %

(Intercept) 4.4742327 2.60714883 8.0603065

FrissonsOUI 0.4652940 0.22863939 0.9565686

FatigueOUI 0.4606066 0.23053471 0.8979129

doulabdoOUI 6.4171705 1.23854612 118.6098571

doulmuscOUI 2.3561507 1.19164951 4.8477539

nezcouleOUI 0.2694029 0.09689153 0.7915805

goutOUI 2.0988779 1.10000173 4.1496464

rassemblementOUI 2.3789337 0.97527920 6.8027416
